# β2 adrenergic agonist attenuates house dust mite-induced allergic airway inflammation through dendritic cells

**DOI:** 10.1186/s12865-014-0039-y

**Published:** 2014-10-30

**Authors:** Go Kato, Koichiro Takahashi, Hiroki Tashiro, Keigo Kurata, Hideharu Shirai, Shinya Kimura, Shinichiro Hayashi

**Affiliations:** Division of Hematology, Respiratory Medicine and Oncology, Department of Internal Medicine, Faculty of Medicine, Saga University, 5-1-1 Nabeshima, Saga, 849-8501 Japan; Institute of Tokyo Environmental Allergy, 1-2-5, Yushima, Bunkyo-ku, Tokyo, 113-0034 Japan

**Keywords:** Bronchial asthma, β2 adrenergic agonist, House dust mite, Dendritic cells

## Abstract

**Background:**

Long-acting β2 adrenergic agonists (LABAs) are commonly used combined with inhaled corticosteroids (ICS) to treat asthmatic patients. Previous reports suggest that LABAs have an anti-inflammatory effect in bronchial asthma, and this should be further investigated. The aim of this study was to investigate whether LABAs inhibit allergic airway inflammation and how this occurs.

**Results:**

We assessed the effect of the LABA formoterol (FORM) on inflammatory cell responses in airway, lung and regional lymph nodes, using an HDM-induced murine allergic asthma model *in vivo*. The effect of FORM on cytokine production from bone marrow derived dendritic cells (BMDCs) stimulated with HDM was evaluated *in vitro*. Adoptive transfer of BMDCs pulsed with HDM in the presence or absence of FORM to naïve mice was performed and the inflammatory response to subsequent HDM challenge was analyzed. FORM treatment suppressed HDM-induced changes and caused an increase in the number of eosinophils and neutrophils in bronchoalveolar lavage. The concentration of IL-4 and IL-17 in lung tissue homogenate was elevated and led to an accumulation of IL-4, IL-13, IL-5 and IL-17 producing cells in regional lymph nodes. FORM inhibited the production of IL-6 and IL-23 from BMDCs stimulated with HDM *in vitro*, and enhanced IL-10 production. The BMDCs adoptive transfer experiment indicated that dendritic cells mediate the effect of FORM, since FORM treatment of BMDCs *in vitro* attenuated airway inflammation.

**Conclusion:**

These results suggested that FORM modulates dendritic cell function and attenuates Th2 and Th17 responses induced by HDM. Thus, we propose that the clinical significance of LABAs should be re-investigated taking into account these immune-modulating effects.

## Background

Bronchial asthma is a chronic airway inflammation associated with inhaled allergens including those produced by house dust mites (HDM) [1,2], and other environmental materials [3]. The pathophysiology of bronchial asthma is characterized by bronchoconstriction, airway hyper-responsiveness, and airway remodeling [4-6]. Although airway eosinophilia is a hallmark of bronchial asthma, the inflammatory process is mediated mainly by Th2 type lymphocytes [7,8]. Recently, evidence has been accumulating that Th17 lymphocytes and their products, such as interleukin-17 (IL-17), mediate neutrophilic inflammation which plays an important role in severe asthma [9,10].

Dendritic cells (DCs) are responsible for the initial antigen-induced immune response, as they act as antigen presenting cells (APC) [11]. In the airways, DCs are present in the subepithelial region and process and present exogenous antigens. Naïve T cells recognize the antigen presented on DCs and differentiate into helper T cells [12,13]. Cytokines produced from DCs determine the direction of helper T cell differentiation and regulate characteristics of the inflammatory response [14]. In this aspect, DCs are a fascinating target for controlling allergic diseases such as bronchial asthma.

Combination therapy with inhaled corticosteroids (ICS) and long acting β2 adrenergic agonists (LABAs) is generally used for the treatment of asthma [15]. The addition of LABAs to moderate doses of ICS has been reported to have greater benefit than double doses of ICS in symptomatic asthmatic patients [16]. Some explanations for this observation have been proposed. Firstly, drug delivery of ICS is improved by LABAs. Secondly, LABAs enhance the function of glucocorticoid receptors in airway smooth muscle cells and epithelial cells [17]. It is also possible that LABAs attenuate inflammatory cell responses [15,18], although the precise mechanism for this requires further clarification.

The function of LABAs is mediated by the β2 adrenergic receptor (ADR) expressed on numerous cell types, including immune cells [19]. The APC activity of Langerhans cells in the skin was suppressed by epinephrine and norepinephrine through β2 ADR [20]. Conversely, Yanagawa and colleagues reported that β2 ADR stimulation enhanced IL-33 production by DCs, and referred to a possible role of β2 agonists in the stress-related progression of Th2-associated disorders [21]. Based on these findings, we aimed to investigate the impact of β2 adrenergic agonists, especially an LABA, on the function of DCs in an HDM-induced allergic airway inflammation model. This is the first report of an LABA significantly suppressing allergic airway inflammation through DCs.

## Methods

### Allergen and chemicals

Two batches of house dust mite (HDM) extract from *Dermatophagoides farina* (Der f) were provided by ITEA Inc. (Tokyo, Japan) as a lyophilized preparation of milled mites. Formoterol (FORM) was provided by Astellas Pharma Inc. (Tokyo, Japan). ADR antagonists used were propranolol for β1 + β2, ICI118,551 for β2, CGP20712A for β1, prazosin for α_1_ and yohimbine for α_2_-ADR respectively (Sigma-Aldrich Co. LLC., St. Louis, MO). Phenylmethylsulfonyl fluoride (PMSF), aprotinin, leupeptin, epinephrine, phorbol 12-myristate 13-acetate (PMA) and ionomycin were purchased from Sigma-Aldrich Co. LLC.

### Mice

Female BALB/c mice (6–8 weeks old, Japan SLC Inc., Hamamatsu Japan) were maintained at the Saga University animal facility under specific pathogen free conditions. Animal experiments were undertaken following the guidelines for care and use of experimental animals of the Japanese Association for Laboratory Animals Science (1987) and were approved by the Saga University Animal Care and Use Committee.

### Protocol for HDM induced airway inflammation model

**Figure 1:**
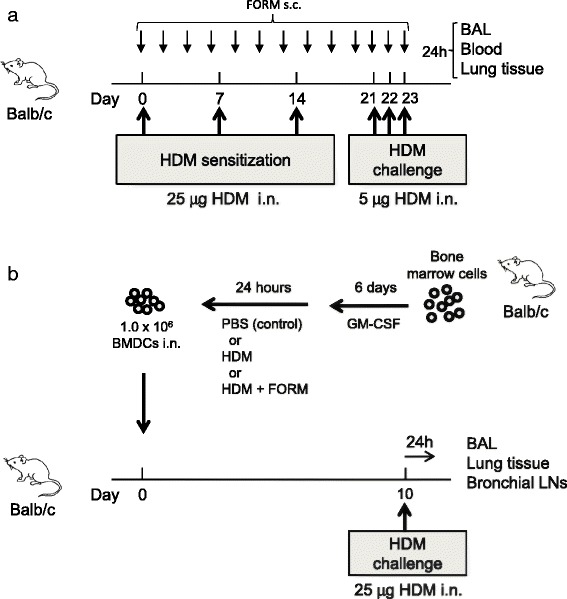
**Protocols for the HDM-induced airway inflammation model. (a)** HDM-induced airway inflammation model. Female BALB/c mice were sensitized three times intranasally with 25 μg HDM at days 0, 7 and 14, and challenged three times intranasally with 5 μg HDM at days 21–23. Twenty-four hours after the final challenge, blood samples, BAL fluid and lung tissues were collected. BALB/c mice receiving PBS at sensitization and challenge were used as controls. **(b)** BM cells were harvested from BALB/c mice. DCs were generated by culturing BM cells with 10% FBS and 10 ng/ml GM-CSF in the culture medium for 7 days. On day 7, DCs were stimulated with HDM or HDM + FORM or PBS (control) for 24 hours. One million DCs were intranasally injected into BALB/c mice on the following day. After 10 days, recipient mice were challenged intranasally with 25 μg HDM. Twenty-four hours after the injection, blood samples, BAL fluid and lung tissues were collected. BALB/c mice receiving unstimulated DCs served as controls.

Mice were inoculated intranasally with 25 μg HDM or vehicle on days 0, 7 and 14. Mice were challenged intranasally with 5 μg HDM on days 21, 22 and 23. On day 24, mice were euthanized by intraperitoneal injection of sodium pentobarbital. Serum, bronchoalveolar lavage (BAL) fluid and lung tissue were harvested for further analysis (Figure 1a). FORM (12.5 ng/animal) was administered subcutaneously three times per week. FORM was dissolved in ethanol 10 mg/ml as a central stock, then was diluted optimal concentration using PBS. On the day when HDM was given, FORM was administered 30 minutes before the HDM inoculation.

### Collection of blood and BAL fluid

Blood and BAL fluid samples were collected as described previously [22]. Briefly, blood was collected by right ventricle puncture. Serum was collected by centrifugation of whole blood at 8,000 × g for 5 min at 4°C and stored at −80°C until needed. Then a 20-gauge tube was inserted into the trachea and the lungs were lavaged twice with 1.0 ml of saline. The cell suspension was centrifuged at 100 × g for 5 min at 4°C. The total number of cells was counted using a hemocytometer. Cytospin samples were prepared from the cell suspension. Cell differentiation was determined by counting at least 300 leukocytes in samples stained with Diff-Quik (Siemens, Germany).

### Histology

Histological examination was performed as previously reported [23]. Lungs were fixed with 10% neutralized buffered formalin (Wako, Osaka, Japan) and embedded in paraffin. Lung sections were stained with Hematoxylin and Eosin (H&E), and periodic acid-Schiff (PAS) stains.

### Preparation of lung homogenate

After bronchoalveolar lavage, the left lung was isolated and homogenized in 50 mM Tris buffered saline (pH 7.4) containing 1 mM EDTA, 1 mM PMSF, 1 μg/ml aprotinin, 1 μg/ml leupeptin, 1 mM Na_3_VO_4_ and 1 mM NaF. The lung homogenates were centrifuged at 10,000 × g for 15 min, and then supernatants were collected and stored at −80°C until required.

### Lymphocyte stimulation in vitro

Mice were inoculated intranasally with HDM or vehicle as described above on days 0, 7 and 14. A bronchial lymph node was removed on day 17, crushed gently and a single cell suspension prepared by passing through a 40 μm nylon mesh (BD Falcon, Franklin Lakes, NJ). Lymphocytes were stimulated with 25 ng/ml PMA and 1 μg/ml ionomycin for 4 hours. After stimulation, supernatants were collected and stored at −80°C.

### Measurement of cytokines by ELISA

IL-4, IL-6, IL-10, IL-13, IL-5, IL-17 and IL-23 were measured using a Quantikine ELISA Kit (R&D systems Inc., Minneapolis, MN) according to the manufacturer’s instructions. All samples were measured in duplicate.

### Preparation of bone marrow derived dendritic cells and in vitro stimulation

Bone marrow (BM) cells were isolated from BALB/c mice as previously reported [24]. BM cells were resuspended at 2.0 × 10^6^ cells/ml in RPMI1640 medium supplemented with 10% fetal calf serum (FCS). The cells were cultured in the presence of 10 ng/ml recombinant murine GM-CSF (R&D systems) at 37°C in a humidified atmosphere containing 5% CO_2_ for 6 days. On day 6, cells were harvested. More than 95% of the cells were positively stained with CD11c, and were used as myeloid dendritic cells (BMDCs) in the following experiments. BMDCs were stimulated with 10 μg/ml HDM, with or without FORM or epinephrine, for 24 hours. BMDCs were pretreated with 1 μM of propranolol, ICI118,551, CGP20712A, prazosin or yohimbine. BMDC supernatants were collected and stored at −80°C.

### Adoptive transfer of BMDCs

As shown in Figure 1b, BMDCs were stimulated with 10 μg/ml HDM, with or without 100 pM FORM for 24 hours. BMDCs were collected and washed twice, and then 1.0 × 10^6^ BMDCs were administered to naïve BALB/c mice intranasally. On day 10, mice were challenged with 25 μg HDM. Twenty four hours later, BAL fluid and bronchial lymph nodes were collected as described above. Lymphocytes were stimulated with 25 ng/ml PMA and 1 μg/ml ionomycin for 4 hours. After stimulation, supernatants were collected and stored at −80°C.

### Measurement of serum Der f specific IgG

To measure Der f-specific IgG, MaxiSorp plates (Nunc, Roskilde, Denmark) were coated with 0.25 μg/ml Der f (Seikagaku co. Tokyo). Plates were washed with 0.1 M phosphate-buffered saline (PBS) containing 0.05% Tween 20. Each well was filled with a blocking solution of 1% BSA (Sigma-Aldrich) in PBS, and incubated for 1 hour. After washing 3 times, 100 μl/well of serum was added and the plates incubated for 1 hour. After washing, IgG1 bound to the plate was detected using biotin-labeled rat anti-mouse IgG1 (Bio-Rad AbD Serotec Ltd, Oxford UK), HRP-streptavidin (Sigma-Aldrich), and 3,3’,5,5’-tetramethylbenzidine (TMB, Invitrogen, CA). The amount of specific antibody was expressed as laboratory units (LU), and samples were compared with a standard serum containing Der f-specific IgG1.

### Statistical analysis

The data are shown as mean ± standard deviation (SD). Analysis of variance (ANOVA) was used for multiple comparison of continuous variables. When there was a significant difference, the difference between each group was tested using Scheffe’s test. All tests were two-sided with a five percent level of significance.

## Results

### Formoterol treatment suppressed HDM-induced airway inflammation

**Figure 2:**
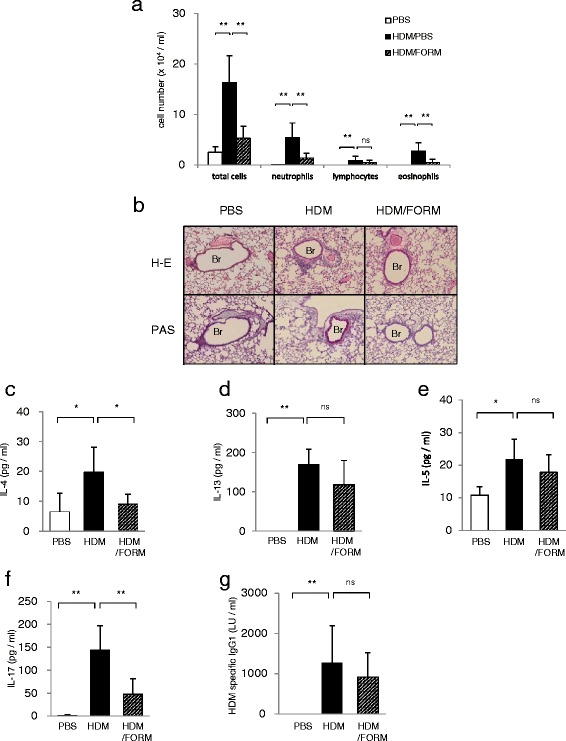
**Formoterol-suppressed HDM-induced airway inflammation. (a)** Total and differential cell counts in BAL fluids. (n = 6 in each group) **(b)** Histological examination of airway inflammation. Sections were stained with H&E (upper panels) or PAS (lower panels). Original magnification was × 200. Br indicates bronchus. Concentrations of **(c)** IL-4, **(d)** IL-13, **(e)** IL-5 and **(f)** IL-17 in the lung tissues were measured by ELISA. (n = 6 in each group) **(g)** Titers of HDM specific IgG1 in the serum are shown. *p < 0.01, **p < 0.05.

We investigated the effect of the β2 adrenergic agonist, FORM, on HDM-induced airway inflammation. Mice were sensitized and challenged with HDM, which resulted in an increase of total cells, neutrophils, lymphocytes and eosinophils in BAL fluid. FORM treatment significantly suppressed BAL cell numbers (Figure 2a). Differences between the two groups were as follows; total number of cells; 16.3 ± 5.3 and 5.3 ± 2.4 × 10^4^/ml (*p* < 0.01), neutrophils; 5.4 ± 2.9 and 1.4 ± 1.0 × 10^4^/ml (*p* < 0.01), lymphocytes; 0.9 ± 0.8 and 0.5 ± 0.4 × 10^4^/ml (*p* = 0.45), and eosinophils; 2.8 ± 1.6 and 0.5 ± 0.6 × 10^4^/ml (*p* < 0.01) in HDM mice and FORM treated mice, respectively. Histological examination showed that inflammatory cell infiltration and goblet cell metaplasia were suppressed by FORM (Figure 2b). The concentrations of IL-4 and IL-17 in lung tissue were significantly decreased in FORM treated mice (Figure 2c, 2f). IL-4 concentration was 19.8 ± 2.9 and 9.1 ± 3.3 pg/ml (*p* < 0.01) and IL-17 was 143.6 ± 63.1 and 47.4 ± 33.5 pg/ml (*p* < 0.01) in HDM mice and FORM treated mice, respectively. There was no difference in the concentration of IL-13 (118.4 ± 61.4 pg/ml in FORM v.s. 168.9 ± 39.8 pg/ml in HDM ), IL-5 (17.8 ± 7.6 pg/ml in FORM v.s. 21.9 ± 6.5 pg/ml in HDM )in lung tissue respectively (Figure 2d, 2e). The titer of serum HDM specific IgG in FORM mice (920.7 ± 600.3 LU/ml) was similar to that in the serum of HDM mice (1,270.0 ± 920.5 LU/ml) (Figure 2g).

### FORM treatment decreased Th2 and Th17 lymphocytes in bronchial lymph nodes

**Figure 3:**
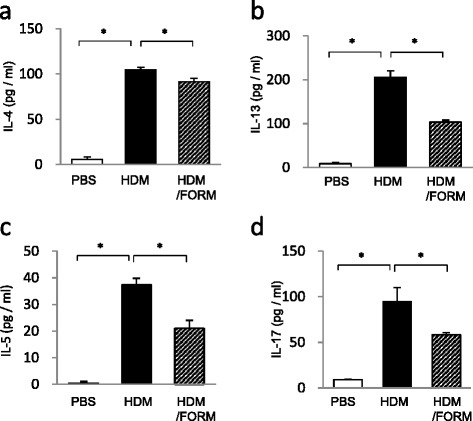
**Formoterol-suppressed accumulation of cytokine producing cells in regional lymph node of the HDM-induced airway inflammation model.** Lymphocytes from bronchial lymph nodes were collected, and cultured at 1.0 × 10^7^/ml in complete RPMI 1640 culture medium containing 10% FBS, in the presence of PMA and ionomycin. After 24 hours, concentrations of **(a)** IL-4, **(b)** IL-13, **(c)** IL-5 and **(d)** IL-17 in the supernatant were measured by ELISA. (n = 6 in each group) *p < 0.01, **p < 0.05.

We next examined whether FORM attenuates the T cell response to HDM in regional lymph nodes. HDM treatment caused an increase in the concentration of IL-4, IL-13 and IL17 in supernatant of lymphocytes (Figure 3). Lymphocytes from FORM treated mice produced significantly less cytokines; IL-4; 104.7 ± 2.8 and 91.5 ± 3.9 pg/ml (*p* < 0.01), IL-13; 205.7 ± 14.3 and 103.6 ± 4.3 pg/ml (*p* < 0.01), IL-5; 37.4 ± 2.5 and 21.1 ± 3.0 pg/ml (*p* < 0.01), and IL-17, 94.7 ± 15.4 and 58.5 ± 2.2 pg/ml (*p* < 0.01), in HDM and HDM/FORM treated mice, respectively. These finding suggest that FORM attenuates the Th2 and Th17 responses to HDM *in vivo*.

To eliminate the possibility that FORM directly affects cytokine production from lymphocytes, lymph node cells harvested from HDM mice were stimulated with PMA and ionomycin in the presence of various concentrations of FORM *in vitro*. As shown in Table 1, FORM concentrations of up to 10,000 pM did not alter IL-4, IL-13 or IL-17 production from lymphocytes.

**Table 1:** **FORM did not have a direct effect on cytokine production from lymphocytes**

**Mice treated with**	**Saline**	**HDM 10 mg/ml**	**HDM 10 mg/ml**	**HDM 10 mg/ml**	**HDM 10 mg/ml**
FORM added *in vitro*	0 pM	0 pM	10pM	100pM	1nM
IL-4 (pg/ml)	3.7 ± 0.5	28.7 ± 2.8	27.2 ± 3.0^#^	28.5 ± 2.0^#^	28.0 ± 2.6^#^
IL-13 (pg/ml)	7.0 ± 15.3	75.1 ± 6.0	72.3 ± 12.5^#^	67.9 ± 14.4^#^	70.8 ± 12.6^#^
IL-17 (pg/ml)	13.2 ± 3.0	41.6 ± 0.3	39.9 ± 2.3^#^	41.6 ± 3.0^#^	40.6 ± 2.1^#^

#There were no significant difference compared with HDM.

### FORM modulated cytokine production from DCs

**Figure 4:**
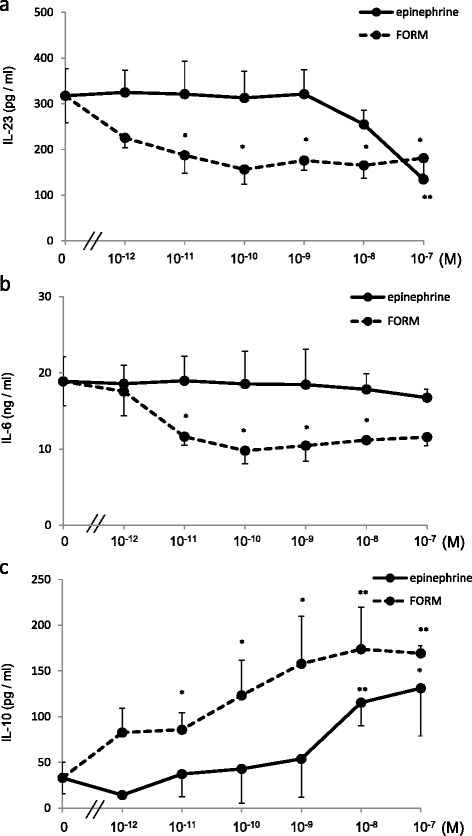
**Formoterol- and epinephrine-attenuated cytokine production from DCs in a β**_**2**_ **adrenergic receptor and dose dependent manner.** BMDCs, 1.0 × 10^6^/ml in complete media, were stimulated by 10 μg HDM in the presence of various concentrations of FORM or epinephrine for 24 hours. Supernatants were collected, and concentrations of **(a)** IL-23, **(b)** IL-6 and **(c)** IL-10 were measured by ELISA. *p < 0.01, **p < 0.05.

We hypothesized that the suppression of T cell responses by FORM *in vivo* was mediated by DCs. We therefore examined the effect of FORM on cytokine production by BMDCs *in vitro*. BMDCs produced IL-23 and IL-6 after HDM stimulation. IL-23 production was suppressed by both FORM and epinephrine in a dose dependent manner (Figure 4a), where the effect of FORM was 10,000 times stronger than epinephrine. IL-6 production was also suppressed by FORM, but not by epinephrine at concentrations of up to 10^−7^ M (Figure 4b). IL-10 production from DCs was enhanced by FORM and epinephrine in a dose dependent manner (Figure 4c).

### The effect of FORM on BMDCs was mediated by β2 ADR

**Figure 5:**
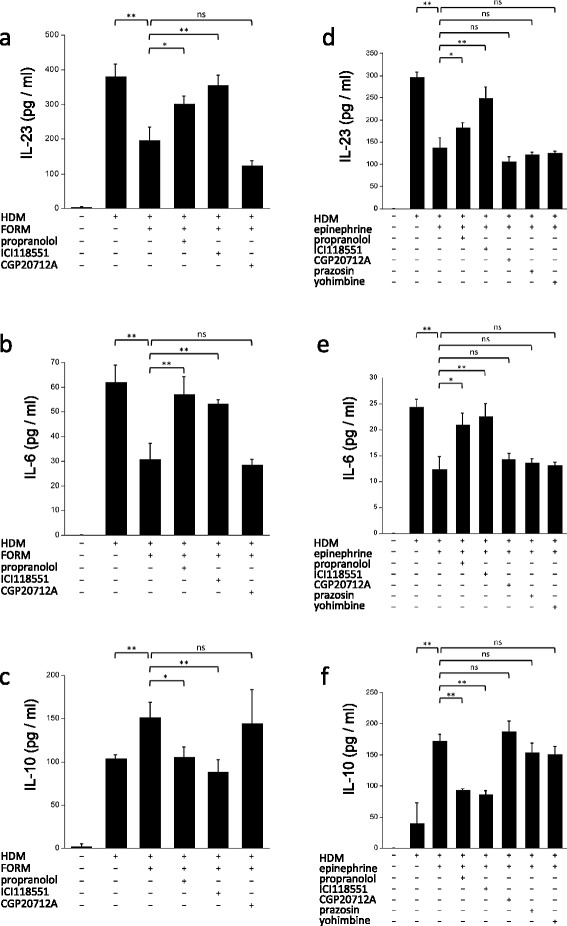
**The effect of FORM and epinephrine on DCs was mediated specifically by the β**_**2**_ **adrenergic receptor.** BMDCs were cultured with 10 μg HDM and 100 pM FORM. The effect of various ADR antagonists on cytokine production was examined, **(a)** IL-23, **(b)** IL-6 and **(c)** IL-10. BMDCs were cultured with 10 μg HDM and 1 μM epinephrine. The effect of various ADR antagonists on cytokine production was examined, **(d)** IL-23, **(e)** IL-6 and **(f)** IL-10. *p < 0.01, **p < 0.05.

We examined whether the effect of FORM is mediated specifically by ADR. To clarify which ADR contribute to the suppression of cytokine production by FORM, specific ADR antagonists used were propranolol for β1 + β2, ICI118,551 for β2, CGP20712A for β1, prazosin for α1 and yohimbine for α2-ADR respectively. IL-23 production from HDM-stimulated BMDCs was suppressed by FORM, 195.2 ± 38.6 pg/ml, but was significantly restored to 301.9 ± 1.8 pg/ml (Figure 5a) when propranolol was added. ICI118,551 also significantly restored IL-23 production to 355.5 ± 29.5 pg/ml, while CGP20712A did not. Propranolol and ICI118,551 restored IL-6 production suppressed by FORM, but CGP20712A did not (Figure 5b). Interestingly, IL-10 production from HDM-stimulated DCs enhanced by FORM, 151.3 ± 17.0 pg/ml, was significantly decreased in a β_2_ ADR pathway dependent manner (Figure 5c).

We used epinephrine, a full agonist for all classes of ADR, to investigate whether the modulation of DC cytokine production is mediated specifically by β2 ADR. The suppression of IL-23 production by epinephrine, 137.4 ± 21.4 pg/ml, was restored, to 182.3 ± 10.7 pg/ml by propranolol, and to 248.0 ± 26.1 pg/ml by ICI118,551. IL-23 production could not be restored by CGP20712A (105.4 ± 11.6 pg/ml), prazosin (121.6 ± 5.5 pg/ml) or yohimbine (124.5 ± 4.5 pg/ml) (Figure 5d). The suppression of IL-6 production by 1 μM epinephrine, 12.3 ± 2.4 ng/ml, was also restored to 20.9 ± 2.3 ng/ml by propranolol, and to 22.6 ± 2.5 ng/ml by ICI118,551. IL-6 production could not be restored by CGP20712A (14.2 ± 1.2 ng/ml), prazosin (13.6 ± 0.7 ng/ml) or yohimbine (13.1 ± 0.6 ng/ml) (Figure 5e). On the contrary, enhanced IL-10 production by epinephrine, 172.0 ± 11.1 pg/ml, was significantly reduced to 92.8 ± 2.7 pg/ml by propranolol and to 86.1 ± 5.9 pg/ml by ICI118,551 (Figure 5f).

### HDM-induced airway inflammation was attributed to cytokines produced by DCs

**Figure 6:**
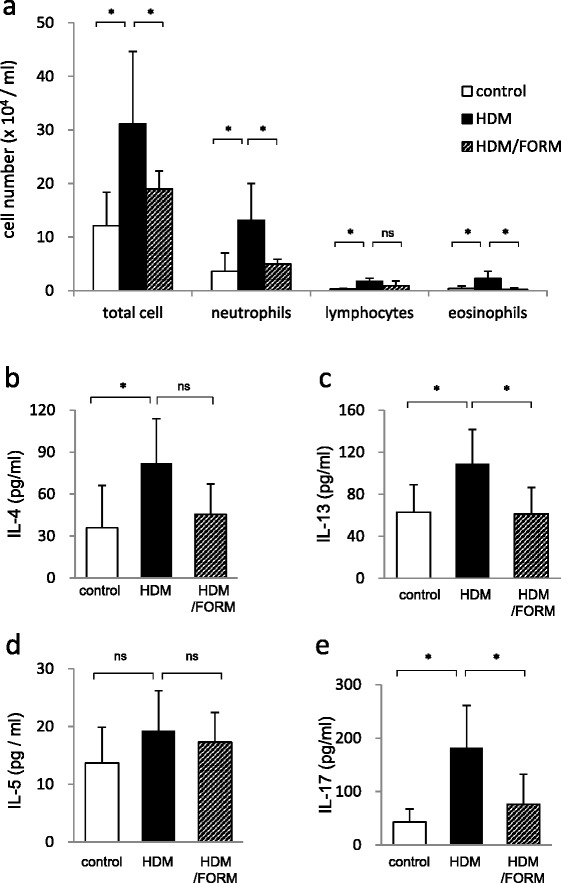
**The effect of Formoterol on the DC adoptive transfer model.** BMDCs were stimulated with 10 μg/ml HDM in the presence or absence of 100 pM FORM for 24 hours. BMDCs were collected and washed twice with PBS, then 1.0 × 10^6^ BMDCs in 50 μl PBS were administered to naïve BALC/c mice intranasally. Control mice were administrated 1.0 × 10^6^ BMDCs without HDM and FORM stimulation (n = 6 in each group). On day 10, mice were challenged with 25 μg HDM. Twenty four hours later, mice were euthanized and BAL fluid and bronchial lymph nodes (LNs) were collected. **(a)** Total and differential cell counts in BAL fluids. After harvesting, bronchial lymph node cells were cultured in the presence of PMA and ionomycin. Concentrations of **(b)** IL-4, **(c)** IL-13, **(d)** IL-5 and **(e)** IL-17 in the supernatant were measured by ELISA. *p < 0.01, **p < 0.05.

To examine whether the effect of FORM on HDM-induced airway inflammation *in vivo* depends on DCs, we performed adoptive transfer of HDM-pulsed BMDCs to naïve mice. When intranasal administration of HDM-pulsed DCs was followed by HDM challenge, the total number of cells (31.2 ± 13.5 × 10^4^/ml), eosinophils (2.3 ± 1.4 × 10^4^/ml) and neutrophils (13.1 ± 6.9 × 10^4^/ml) in BAL fluids was significantly increased compared with mice given sham-pulsed DC and HDM challenge (Figure 6a). BAL cell counts in the latter group were total cells; 12.1 ± 6.3 × 10^4^/ml, eosinophils; 0.4 ± 0.4 × 10^4^/ml, and neutrophils; 3.6 ± 3.4 × 10^4^/ml. When DCs pulsed with HDM in the presence of 100 pM FORM *in vitro* were administered to mice, the BAL cell numbers were significantly decreased in comparison to HDM group; total cells; 19.0 ± 3.3 × 10^4^/ml (*p* < 0.05), eosinophils; 0.3 ± 0.3 × 10^4^/ml (*p* < 0.05), and neutrophils; 5.0 ± 0.9 × 10^4^/ml (*p* < 0.05), respectively (Figure 6a).

Mice given HDM-pulsed DCs and antigen challenge showed lymphocyte responses similar to mice directly sensitized with HDM. The concentration of IL-4, IL-13, IL-5 and IL-17 in lymph node cell culture was 82.1 ± 31.8 pg/ml, 108.9 ± 32.8 pg/ml, 19.3 ± 6.9 pg/ml, and 181.7 ± 79.6 pg/ml, in the HDM-pulsed DC group, and 35.9 ± 30.3 pg/ml, 63.0 ± 26.2 pg/ml, 13.7 ± 6.2 pg/ml, and 43.1 ± 24.6 pg/ml, in the sham-pulsed DC group, respectively (Figure 6b-e). Significant differences between the two groups were found for levels of IL-4 (*p* < 0.05), IL-13 (*p* < 0.05) and IL-17 (*p* < 0.05). When DCs were pulsed with HDM in the presence of FORM, the concentration of IL-4, IL-13, IL-5 and IL-17 in lymph node cell culture supernatants decreased to 45.6 ± 21.6 pg/ml, 61.4 ± 24.9 pg/ml, 17.3 ± 5.1 pg/ml, and 76.3 ± 56.2 pg/ml, respectively. The difference between the HDM group and the HDM/FORM group was significant for levels of IL-13 and IL-17 (*p* < 0.05).

## Discussion

In this study we demonstrated that FORM suppressed both HDM-induced accumulation of inflammatory cells in the airway and goblet cell metaplasia of airway epithelium in mice. These effects were associated with decreased IL-4 and IL-17 in lung homogenate and decreased accumulation of Th2- and Th17-cytokine production in regional lymph nodes. These observations raise the possibility that β2 adrenergic agonists can attenuate response of DCs to HDM and thus lead to modulation of T cell function.

DCs contribute to the initial step of an allergic response after environmental exposure, and play a key role as APC in helper T cell responses [11]. DCs induce naïve lymphocytes to differentiate into Th2 or Th17 cells through production of IL-6 and IL-23 [25,26]. Recent evidence has shown that catecholamines can regulate the immune response through DCs. Antigen presentation by Langerhans cells in the skin was suppressed by epinephrine and norepinephrine in a β2 ADR dependent manner [20]. Crosstalk between TLR4 and β2 ADR transduction pathways in DCs was demonstrated *in vitro* and *in vivo* [27]. Matsushita and colleagues reported that dopamine D1-like antagonists attenuate the Th17-mediated immune response through DCs and airway inflammation [28]. Therefore, we hypothesized that a β2 ADR agonist has inhibitory effects in allergic airway inflammation.

We demonstrated that FORM suppressed IL-6 and IL-23 production from BMDCs *in vitro* and that production was restored by treatment of DCs with β2 ADR antagonists. Furthermore, epinephrine, a full agonist for α and β ADRs, also suppressed cytokine production, which was restored by β2 selective ADR antagonists but not by other classes of ADR antagonists. These observations suggest that the effect of FORM and epinephrine on BMDC is specific and mediated by the β2 ADR signaling pathway. The effect of FORM was about 10,000 times stronger than that of epinephrine which may be explained by the nature of FORM as a long acting β2 agonist. FORM initially diffuses into the plasma membrane of cells, and then is slowly released from the cell, where it can come into contact with β2 ADR [29]. Interestingly, IL-10 production from DCs was enhanced by both FORM and epinephrine. IL-10 is known as an inhibitory cytokine in allergic airway inflammation [30]. Enhanced IL-10 production in DCs contributes to self-limiting airway inflammation. Taken together, these data indicate that a β2 ADR agonist can modulate the function of HDM-stimulated BMDC and suppress allergic inflammation.

A recent study reported that repeated challenge with methacholine caused bronchoconstriction without additional airway inflammation and airway remodeling in asthmatic patients [31]. This finding raises a question that FORM suppressed bronchoconstriction and thus inhibits lymphocyte reactions. To address this question, we conducted a BMDC adoptive transfer experiment. The treatment of BMDCs by FORM suppressed airway inflammation and accumulation of Th2/Th17 cytokine producing lymphocytes in regional lymph nodes. These results strongly suggest that β2 ADR stimulation can attenuate inflammatory processes in an HDM-induced airway inflammation model, through modulation of DC function.

The effect of ICS on airway inflammation and hyper-responsiveness is well established. Nevertheless, there remain some issues to be solved. Firstly, a number of patients have airway inflammation resistant to ICS. Bronchial asthma is a heterogeneous disease with several proposed clinical phenotypes [32]. Among them, IL-4 and IL-13-mediated severe atopic asthma with elevated serum IgE and periostin can be treated effectively with an anti-IgE monoclonal antibody, omalizumab, or an anti-IL-13 monoclonal antibody, lebrikizumab [33-36]. Severe non-atopic eosinophilic asthma of late onset is often associated with chronic sinusitis and nasal polyps. This disease is resistant to high dose ICS but responsive to systemic corticosteroids and anti-IL-5 therapy [32,37]. Evidence suggests the involvement of the IL-5/IL-33 signaling pathway in the pathogenesis of this phenotype [38,39]. Other asthmatic patients have corticosteroid resistant neutrophil-dominant disease. IL-17 has been shown to be involved in the pathogenesis of neutrophil-dominant asthma [9,40,41]. Although macrolide antibiotics may be effective in this steroid-resistant condition [42,43], a better solution is required. Our finding that a long acting β2 receptor agonist, FORM, modulates the Th17 response suggests another possible solution.

A second issue is that ICS treatment has to be continued in order to maintain a well-controlled state. In most asthmatic patients, ICS strongly suppresses airway inflammation and establishes a symptom free condition. However, ICS cannot modulate the mechanisms underlying the pathogenesis of asthma, such as atopic sensitization. Thus, once ICS is discontinued, airway inflammation gradually recurs. Asthmatic patients frequently ask their physicians how long they should continue ICS treatment once they have become free of symptoms. To best answer this question, treatment that can improve the underlying condition should be established. Immune-modulation therapies, including antigen desensitization, have been applied to asthmatic patients with modest success [44]. In animal models of airway inflammation, autoimmune encephalomyelitis, nephrotoxic serum nephritis, and rheumatoid arthritis, an antagonist to the dopamine receptor has been reported to attenuate DC function and thus the immunological response [28,45-47]. Our findings extend this concept and indicate for the first time that an LABA can directly suppress the immunological response *in vivo*.

ICS/LABA combination therapy is a widely used and reliable treatment for asthmatic patients [48,49]. However, recent studies have raised concerns about the safety of LABAs in asthma treatment, due to evidence of an increased risk of severe exacerbation of asthma symptoms leading to hospitalization in pediatric and adult patients, as well as some fatalities [50,51]. Accordingly, the U.S. Food and Drug Administration announced in February 2010 that LABAs should not be used alone in patients with asthma, and that they should be used for the shortest duration of time required achieving control of asthma symptoms [52].

## Conclusion

Our findings suggested that FORM modulates dendritic cell function and attenuates Th2 and Th17 responses induced by HDM. Thus, we propose that the clinical significance of LABAs should be re-investigated taking into account these immune-modulating effects.

## Abbreviations

HDM: House dust mite
Der f: Dermatophagoides farinae
LABA: Long acting beta 2 adrenergic agonist
ADR: Adrenergic receptor
ICS: Inhaled corticosteroid
FORM: Formoterol
BMDC: Bone marrow derived dendritic cell
APC: Antigen presenting cells
BAL: Bronchoalveolar lavage
H&E: Hematoxylin and Eosin
PAS: Periodic acid-Schiff
